# Impact of mass distribution of long-lasting insecticide nets on the incidence of malaria in Lomami, Democratic Republic of Congo: a study based on electronic health record data (2018 - 2019)

**DOI:** 10.11604/pamj.2023.45.89.33099

**Published:** 2023-06-20

**Authors:** Theddy Kazadi Kabeya, Jean Claude Musasa Kasongo, Nathan Bukasa Matumba, Damien Ilunga Tshibangu, Luis Ayerbe Garcia-Morzon, Eduardo Burgueño

**Affiliations:** 1 School of Public Health, University of Mwene Ditu, Lomami, Democratic Republic of Congo,; 2 Health Regional Division, Kabinda, Lomami, Democratic Republic of Congo,; 3 Mwene-Ditu Health Zone, Lomami, Democratic Republic of Congo,; 4 Centre of Primary Care, Queen Mary University of London, London, United Kingdom,; 5 Centre Médical Vésale, Ngaliema, Kinshasa, Democratic Republic of Congo,; 6 School of Medicine, Official University of Mbujimayi, Kasai-Oriental, Democratic Republic of Congo

**Keywords:** Impact, long-lasting insecticide nets (LLIN), malaria incidence, Lomami

## Abstract

**Introduction:**

holoendemic, malaria remains one of the major public health problems in Lomami Province in the Democratic Republic of Congo (DRC). To fight against it, a free mass distribution of long-lasting insecticide nets (LLINs) was organized in July 2019 throughout the province. The present study aimed to assess the incidence of malaria and its impact on anaemia of children from 0 to 59 months in this region before and after this intervention.

**Methods:**

we had conducted a retrospective observational study from June to December 2018 and June to December 2019. The data were collected on District Health Information System version two (DHIS2) and analyzed with T-tests to compare the incidence rates before (second semester 2018) and after the distribution of LLINs (second semester 2019).

**Results:**

the evolution of malaria cases immediately dropped after the distribution campaign. The incidence rates per 1,000 inhabitants in 2018 and 2019 were 106 and 107 respectively in the general population; 302 versus 305 in children aged 0 to 59 months and 219 versus 209 in pregnant women. The differences in incidence were not statistically significant with p values 0.497, 0.4602, and 0.3097 respectively. However, it was observed that the decrease in malaria cases led to a decrease in anaemia cases in general.

**Conclusion:**

the LLIN distribution campaign did not decrease the incidence of malaria. The synergy of preventive interventions to reduce the incidence of malaria remains key.

## Introduction

Malaria is a public health problem in the world [1]. It is the leading cause of consultation and hospitalization in certain countries of sub-Saharan Africa and in the DRC in particular [2,3]. It is a frequent cause of anaemia in children under five and therefore responsible for a large number of deaths recorded in this age category [4-7]. It is also a health issue that results in the impoverishment of many families [8]. In addition, it can have repercussions on pregnant women causing miscarriages, premature deliveries, low birth weight, and maternal deaths from anaemia [1,9].

Global epidemiological data from 2018 indicated that the incidence of malaria fell from 71 to 57 cases per 1,000 inhabitants from 2010 to 2018 with a slowdown in the last 5 years. In the World Health Organization (WHO) Africa Region, 213 million cases were recorded, or 93% of all cases. About 85% of the total number of malaria cases and deaths worldwide came from 19 countries in sub-Saharan Africa and India and 94% of all deaths were from the Africa Region [1].

The DRC occupies the second place with 12% of cases after Nigeria (25%) among the six countries which alone have recorded nearly half of the global cases [1]. About 97% of its population is exposed to this endemic with nefarious consequences [3,6,9,10]. In addition, it leads to catastrophic costs: $ 10 for the treatment of uncomplicated malaria and $ 198.3 for severe malaria [3]. In Lomami Province, epidemiological surveillance data for the past four years show that malaria ranks first among diseases with epidemic potential in terms of morbidity and mortality, especially for children under five according to District Health Information System version 2 (DHIS2).

Malaria prophylaxis can be individual for those at risk. In places where it is endemic, one can use: intra-home spraying, promotion of sanitation, use of LLINs, preventive chemotherapy for pregnant women (intermittent preventive treatment), infants and seasonal malaria chemoprevention (seasonal malaria chemoprevention) [4,11-14]. Indoor spraying of insecticides gives good results [15] but with the risk of resistance to the chemicals used [8,16]. The cost can be exorbitant for poor families [11,17]. Chemoprophylaxis in DRC is based on sulfadoxine-pyrimethamine (SP) and is only for pregnant women. Cases of resistance to this drug have already been reported [2,5,18-20].

The main intervention in malaria prevention is the use of LLINs [9,21-24]. It significantly reduces the mortality rate to around 50% [2,6,23]. WHO recommends organizing the distribution of LLINs either as a mass campaign, in schools every 3 years in areas with a high malaria endemicity such as Lomami Province because its effectiveness gradually decreases over the 3 years after distribution [5,24,25].

In view of the better results obtained elsewhere following the mass distribution of LLINs, the DRC has also opted for it as an intervention to be carried out every 3 years. In Lomami Province, the first LLIN distribution campaign took place in 2011, then the second in 2014 and the most recent in July 2019 taking into account the 16 Health Zones (HZs) that make it up with a coverage rate of 99% household. This intervention requires the mobilization of a lot of financial and logistical resources, not to mention human resources [22]. However, the morbidity of malaria in Lomami Province remains high despite this distribution. Therefore, this study wants to assess the incidence of malaria before and after the mass distribution of LLINs and his impact on anaemia of children from 0 to 59 months to provide the evidence that is lacking in this area.

## Methods

**Study design and setting:** we conducted a retrospective observational study from June to December 2018 and June to December 2019 in Lomami Province. It is one of 26 provinces that make up the DRC, located in the center at coordinates 6° 08' 01'' south and 24° 29' 01'' east with an area of 56,426 km^2^divided into 16 Health Zones (HZ) with a population of 3.996.040 inhabitants. The year 2018 was used as a baseline. All 16 HZ making up Lomami Province were included in this study as all participated in the last mass distribution of LLINs in July 2019.

**Participants, variables and study size:** exhaustive sampling was used in this study. It included the general population, specifically children from 0 to 59 months and pregnant women who had suffered and consulted for malaria. The numbers of malaria cases in the population, in children aged 0 to 59 months, pregnant women, and cases of anaemia in children 0 to 59 months were the outcome variable for this study.

**Data sources, bias, quantitative variables, and statistical methods:** the data analyzed in this study were taken from DHIS2 (basic service, consultation data), which is the national health data management software that Lomami Province started using since 2016. Poor data completeness and coding errors on the DHIS2 can lead to bias and influence the results. However, DHIS2 standards state that when data completeness is 80% or more, analyzes can be done. The collected data are malaria cases (confirmed by a rapid diagnostic test or thick film smear) and cases of anaemia (according to low levels of haemoglobin). These were then exported to Microsoft Excel for the development of figures and tables, and an analysis and interpretation of the results. First, the incidence rates were calculated for the period before the mass distribution of LLIN (second half of 2018). The same exercise was done for the year 2019, then the results of two periods i.e. the second semesters of two years were compared with particular regard to the period after the distribution of LLINs (from July 2019). These parameters were calculated for the whole province on the one hand and for each one of the HZs on the other hand. T-tests were used to find the difference between the incidence of malaria in the whole population, and also in different sociodemographic and clinical categories in 2018 and 2019. The p-value was calculated with the function T-test of Microsoft Excel. The significance level was set at p<0.05.

## Results

The data analyzed concern all adults who have suffered from malaria, but also children aged 0 to 59 months and pregnant women. Cases of anaemia only concerned children from 0 to 59 months.

**Figure 1:**
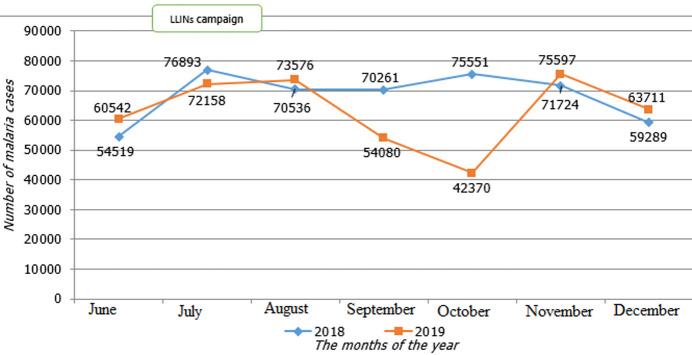
evolution of the number of malaria cases per month in the second semester 2018 and 2019

The monthly evolution of malaria cases (Figure 1) in Lomami Province showed that after the distribution of LLINs, from July 2019, the number of malaria cases was lower than in 2018 in September and October respectively 54,080 and 42,370 against 70,261 and 75,551 cases.

**Figure 2:**
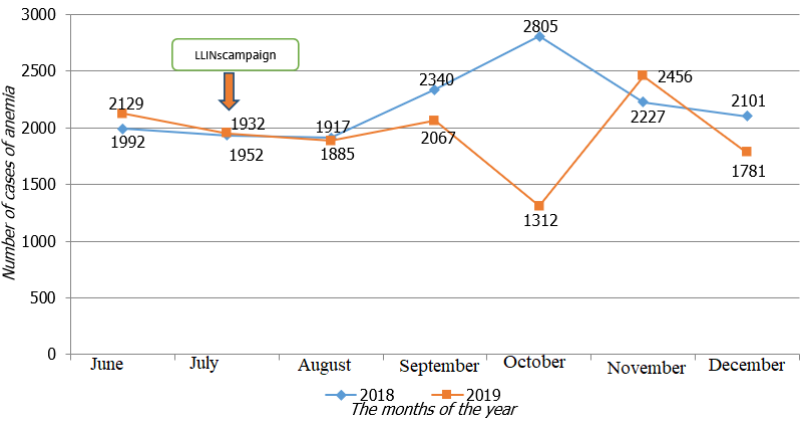
number of cases of anaemia in children aged 0 - 59 months per month in second semester 2018 and 2019

In general, the monthly trend of all cases of anaemia in children aged 0 to 59 months in the provinces experienced a decline from July 2019 after the distribution of LLINs in particular 1885 against 1917 in August, 2067 against 2340 in September, 1312 against 2805 in October and 1781 against 2101 in December 2019 (Figure 2).

**Figure 3:**
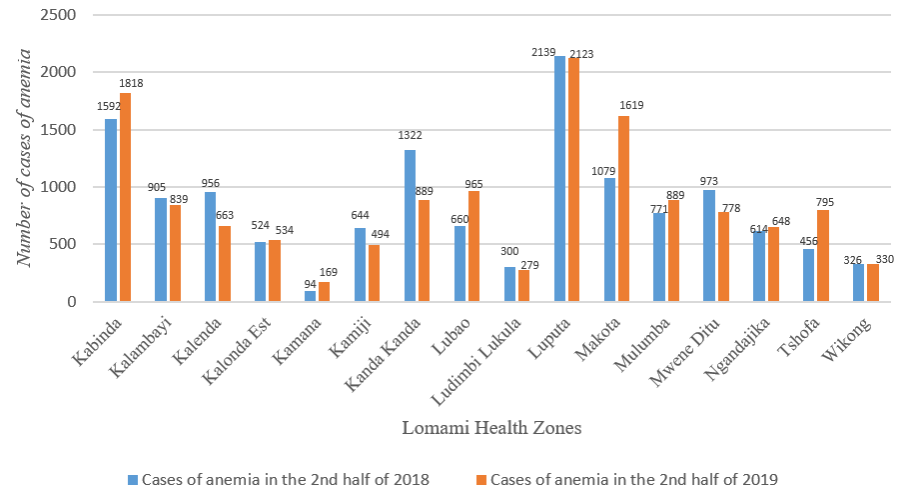
number of cases of anaemia in children aged 0 to 59 months by health zone in the second semester in 2018 and 2019

The comparison of anaemia cases recorded between 2018 and 2019 in the different HZs showed disparities. On the one hand, there were HZs whose number of cases had declined and on the other hand, those whose cases had increased (Figure 3).

Overall, malaria incidence as cases per 1,000 individuals vary from one HZ to another. In the general population, they were respectively 106 in 2018 and 107 in 2019, p = 0.497 (Table 1). In children 0 to 59 months, we observed 302 in 2018 and 305 in 2019, p = 0.46 (Table 2). Among pregnant women, 219 in 2018 and 209 in 2019; p = 0.3097 (Table 3).

**Table 1:** malaria incidence rate by health zone in the second semester in 2018 and in 2019 in the population

Health zone	Incidence rate p 1000 in 2018	Incidence rate p 1000 in 2019
Kabinda	121	119
Kalambayi	188	162
Kalenda	137	110
Kalonda Est	96	65
Kamana	94	119
Kamiji	120	84
Kanda Kanda	175	128
Lubao	49	130
Ludimbi Lukula	62	105
Luputa	107	113
Makota	159	147
Mulumba	42	58
Mwene Ditu	144	126
Ngandajika	74	82
Tshofa	86	93
Wikong	70	84
Total province	106	107

T student = - 0.0077; p = 0.497

**Table 2:** malaria incidence rate by health zone in the second semester in 2018 and in 2019 in children aged 0 - 59 months

Health zone	Incidence rate p 1000 in 2018	Incidence rate p 1000 in 2019
Kabinda	389	372
Kalambayi	539	480
Kalenda	423	332
Kalonda Est	271	189
Kamana	262	330
Kamiji	305	238
Kanda Kanda	543	392
Lubao	132	357
Ludimbi Lukula	149	258
Luputa	325	338
Makota	467	431
Mulumba	104	158
Mwene Ditu	351	305
Ngandajika	218	265
Tshofa	204	233
Wikong	170	211
Total province	302	305

T student = - 0.1016; p = 0.46

**Table 3:** malaria incidence rate by health zone in the second semester in 2018 and in 2019 among pregnant women

Health zone	Incidence rate p 1000 in 2018	Incidence rate p 1000 in 2019
Kabinda	205	218
Kalambayi	437	375
Kalenda	288	231
Kalonda Est	219	146
Kamana	124	162
Kamiji	314	209
Kanda Kanda	284	235
Lubao	141	281
Ludimbi Lukula	129	209
Luputa	224	211
Makota	349	313
Mulumba	160	109
Mwene Ditu	241	253
Ngandajika	157	139
Tshofa	127	141
Wikong	148	188
Total province	219	209

T student = 0.5072; p = 0.3097

As mentioned above, the low completeness of the data can bias the results. However, in this study, data completeness in DHIS was 95.5% in 2018 and 98.5% in 2019, respectively. This allows us to analyze this data.

## Discussion

The distribution of LLINs had led to a decrease in malaria cases in July but particularly in September and October 2019. Malaria incidence rates in the general population, in children 0 to 59 months and pregnant women vary from one health zone to another. After the T-test, the p-value was above the normal threshold in these three groups. The monthly evolution of all cases of anaemia in children aged 0 to 59 months had fallen from July 2019 after the distribution of LLINs, except for the increase in November 2019. A comparison of cases of anaemia recorded between 2018 and 2019 in the different HZ showed disparities.

The data analyzed in this study was taken from DHIS2, an electronic database that the province has started using since 2016. As this data is online, it can be checked by anyone regardless of location. The completeness of the data was acceptable over the period considered in this study during our consultation of DHIS2. Apart from mass distribution, there is also the distribution of LLINs during prenatal consultation, preschool consultation and in schools which is done with the possibility of having a positive impact on the decrease in the incidence of malaria. There was also no particular situation such as climate change that would influence the results.

The decrease in malaria cases observed in the months following the free distribution of the LLIN, particularly in September and October 2019 is justified by the following: immediately after the campaign, the population still has fresh notions of efficiency and effective use of the LLIN because the campaign is preceded by several awareness sessions. The population uses LLIN correctly and the insecticides used for impregnation are still effective. However, the concern relates to the recrudescence of cases in November, with a slight decrease in December of the same year although it remained higher than the number of cases of the year 2018. It is an ephemeral effectiveness (2 months) whereas several studies carried out around the world show that the rate of effectiveness of LLIN after its distribution was 3 years [5,24,26]. Concomitant use of old and new LLINs may be the cause. Because the protection offered by the old LLINs will only be mechanical and not insecticidal, which also reduces the number of plasmodia in the environment. This concomitant use of old and new LLINs in DRC was also noted in the Multiple Indicator Cluster Survey [27]. LLINs that can become vulnerable due to the number of holes they have can only be a hypothesis to be raised as a last resort after a long period of use of more than at least 4 to 5 years [28].

Certainly, several studies demonstrate the effectiveness of LLINs in preventing malaria in the world [9,21-24] and in DRC [29]. The comparative analysis of the incidence rates between the two periods shows that there is no statistically significant difference. In other words, the LLIN distribution campaign did not really reverse the epidemiological situation. In our opinion, this is linked to the fact that prevention in our environment tends to focus only on the use of LLIN.

The disparities observed in the HZ are linked to certain uncontrolled risk factors. A slight decrease in the incidence of malaria was observed in 2019 among pregnant women, although not statistically significant. It would be linked to the combination of two other interventions outside the mass distribution of LLINs; namely the distribution of the latter during the prenatal consultation and the intermittent preventive treatment. In view of these data, we are of the opinion of those who demand the simultaneous combination of several preventive methods [2,15,23,30] because our results corroborate with those of Chaccour in Mozambique [17], Soussa [23] in Brazil, and Lemoine [31] in Haiti to name a few.

The evolution of the curve of anaemia cases is superimposed on that of malaria. It is fair to say that the slight decrease in cases of malaria also reduced cases of anaemia in children because this pathology is the main cause of anaemia alongside acute malnutrition and sickle cell anaemia in our community [10]. Nevertheless, we noted a few exceptional health zones (Kabinda, Makota) where there was a decrease in malaria cases but an increase in anaemia cases. In our opinion, the other causes of anaemia in children would have occurred concomitantly with malaria in these HZs.

The elimination of malaria as a public health problem must simultaneously combine preventive interventions: peri and intra-domiciliary sanitation, environmental sanitation for the destruction of larval breeding grounds, chemoprophylaxis, intra-domiciliary spraying, and use of effective and correct LLINs. In view of the above, the question of this upsurge just two months after distribution remains unresolved and requires further study on the effective use of LLINs by the population of Lomami Province on the one hand and on the other hand, the continuation of this study to observe the trend of the curve in the coming months.

**Limitation:** this study was based primarily on data from DHIS2. Poor data completeness and coding errors on the DHIS2 can lead to bias and influence the results.

## Conclusion

The LLIN distribution campaign as a preventive intervention for malaria in Lomami Province did not lead to a statistically significant decrease in the incidence rate between the second semesters of 2018 and 2019 in the general population, in children aged from 0 to 59 months, nor in pregnant women. Globally, cases of anaemia in children 0-59 months are decreasing along with the decline in malaria incidence. However, the observation of the adverse effect in certain HZs, the existence of high incidence rates and cases of anaemia despite the free distribution campaign, it should be emphasized that the promotion should not only be limited to the use of LLIN but to the combination of all related preventive measures. In the future, it is desirable to continue with this study to determine the epidemiological profile of malaria in the months and years to come in order to verify the theory of the effectiveness of LLIN in the 3 years after distribution but also to identify the determinants of its endemicity in Lomami Province.

### What is known about this topic

- *The use of LLINs protects against malaria*;
- *The effectiveness of LLINs after the distribution is approximately 3 years after the distribution*.

### What this study adds

- *The prevention of malaria in rural areas can only be effective if there is a combination of several preventive strategies and not only focused on the promotion of LLINs*;
- *The monthly trend of anaemia cases in children 0-59 months outperforms that of malaria in the same age group*.
